# Frequency Noise Suppression of a Single Mode Laser with an Unbalanced Fiber Interferometer for Subnanometer Interferometry

**DOI:** 10.3390/s150101342

**Published:** 2015-01-12

**Authors:** Radek Šmíd, Martin Čížek, Břetislav Mikel, Ondřej Číp

**Affiliations:** Institute of Scientific Instruments, v.v.i., Academy of Science of Czech Republic, Královopolská 147, Brno 61264, Czech Republic; E-Mails: cizek@isibrno.cz (M.C.); mikel@isibrno.cz (B.M.); ocip@isibrno.cz (O.C.)

**Keywords:** unbalanced interferometer, fiber spool, PI control, frequency noise

## Abstract

We present a method of noise suppression of laser diodes by an unbalanced Michelson fiber interferometer. The unstabilized laser source is represented by compact planar waveguide external cavity laser module, ORION^TM^ (Redfern Integrated Optics, Inc.), working at 1540.57 nm with a 1.5-kHz linewidth. We built up the unbalanced Michelson interferometer with a 2.09 km-long arm based on the standard telecommunication single-mode fiber (SMF-28) spool to suppress the frequency noise by the servo-loop control by 20 dB to 40 dB within the Fourier frequency range, remaining the tuning range of the laser frequency.

## 1. Introduction

Generation of the length of etalons and measurement of the lengths of passive Fabry-Perot cavities [1,2] or their displacement [3,4] is limited by the vibration of mirrors, thermal fluctuations, speed of lock-loops and by noise. Although the linewidth of the laser source is not a limiting factor in the case of a narrow linewidth laser, we should better observe and understand the cavity behavior with a noise-free laser. The laser linewidth could be stabilized directly to the mode of the passive ultrastable cavity, but the tunable range of the stabilized laser source is limited to the tuning range of the length of the ultrastable cavity. The best way to track the cavity length changes is to pre-stabilize the laser linewidth with a noise-free method. The typical external laser cavity has a planar-concave or a confocal configuration with a mirror distance of 100 mm or 150 mm. The finesse of the cavity is usually over 2000 and in atomic clock applications can achieve up to 100,000 s [5]. The linewidth of the typical cavity mode then reaches below kHz and MHz, which correspond to pm to nm uncertainty in the cavity length. The measurement of the displacement of the Fabry-Perot cavities thus could be made by a tunable laser with sub-kHz to sub-MHz linewidth with an optical reference laser with better or the same linewidth [6,7]. The He-Ne lasers can achieve 10^−13^ [8–10] stability, but the PZT tuning range limits the tunability only to 1 GHz. The typical diode lasers have larger tunability, but the linewidth of 30 MHz is suitable only for cavities with very low finesse. The distributed feedback lasers (DFB) [11] can achieve roughly about 1 MHz linewidth corresponding to 10^−8^ relative uncertainty of measurement and 1 nm uncertainty for a 100 mm-long cavity, but offer a wide tunable range of nms or hundreds of GHz. Better linewidth can be achieved for external cavity laser (ECL) diodes based on DFB lasers and grating [12,13], but these complex laser systems may be quite large and they are sensitive to alignment and introduce power losses. The state-of-the-art ECLs include a planar waveguide with the fiber Bragg grating and reach linewidths under 3 kHz [14], but the tuning range usually covers only tens of pm, i.e., a few GHz. Further improvement of relative uncertainty can be achieved by laser frequency noise suppression. The frequency noise suppression can be achieved by locking the laser to an absorption line of a spectroscopic gas cell [6,15] or to an ultrastable cavity made from very low expansion material, such as ultra-low expansion (ULE) glass [16–20]. The typical methods are the 1^st^-derivative technique [21,22] or the Pound-Drever-Hall technique [23], and they can reach useful linewidths, but their main limitation is in tuning range of the lasers. Measurement with a narrow linewidth laser is limited by the laser frequency noise. Frequency noise practically limits the precision of the displacement measurement. The suppression of the frequency noise of the laser maintaining the wide laser wavelength tuning range can be achieved by methods using an unbalanced fiber interferometer [24,25]. In this work, we present a frequency noise suppression of the ORION^TM^ laser module [14] using an unbalanced Michelson fiber interferometer and servo-loop control. The method can be used to monitor the displacement of the Fabry-Perot cavity with sub-nm uncertainty and with very high displacement measurement precision.

## 2. Method and Set-Up

### 2.1. Principles of the Method

The method of frequency noise suppression and narrowing of laser linewidth in the servo loop with the fiber interferometer is based on the fact that two arms of interferometers are not of the same length, and the light mixed in the splitter contains the information about the delay between the two arms. One can find an extensive explanation of theory of the linewidth measurement in [24,25]. In this work, we will present only the most important equations and notes. Let us call the longer arm of the interferometer *L*_1_ (*L*_1_ ≈ 1 km) and the shorter arm *L*_2_ (*L*_2_ ≈ 1 m). The unbalanced interferometer causes the delay *τ* between the two arms of the interferometer. Thus:

(1) $$\tau =\frac{n_{g}\cdot \left(L_{2}-L_{1}\right)}{c}$$

where *c* is the speed of light and *n_g_* is the refractive index of the fiber in the interferometer. The typical optical fiber for the infra-red region (SMF-28 [26]) consists of a fused silica core with the refractive index *n_g_* = 1.4682. On the output of the interferometer, we detect the beat note signal between the laser frequency propagating in the long and the short arm. The detected phase difference Φ(*t*) = Φ_0_ + *δ*Φ(*t*) with phase fluctuation *δ*Φ(*t*) corresponds to the frequency fluctuation of the laser *δν*(*t*). The transfer function of the interferometer is defined by the Fourier transform of phase fluctuation *δ*Φ̃(*f*) and laser frequency fluctuation *δν̃*(*f*) [25,27]:

(2) $$T\left(f\right)=\frac{\delta \tilde{\Phi }\left(f\right)}{\delta \tilde{\nu }\left(f\right)}=\frac{1-e^{-\text{i}2\pi f\tau }}{\text{i}f}\left[\text{rad}\cdot {\text{Hz}}^{-1}\right]$$

where *f* is the Fourier frequency and *τ* is the time delay between two interferometer arms. *T*(*f*) is a Fourier product of the interference signal beat note frequency fluctuations Δ*ν* between the arms delayed by *τ* [28]. The complex coefficient *i* denotes to the frequency (real part) and phase (imaginary part) noise transfer function of the system.

The interferometer will not pass certain frequencies. They are defined by *T*(*f*) = 0. From Equation (2), we can write:

(3) 2πτ⋅f=2kπ

where *k* is the integer multiple and *f* is the Fourier frequency. Then, for each *f*_k_ = *k*/*τ* → *T*(*f*_k_) = 0. A control system over a large frequency bandwidth (*k* > 1) is feasible [27], but experimentally, the most practical is the bandwidth *f ϵ*(0, *f*_1_) for *k* = 1, where *f*_1_ = 1/*τ*.

There are two common types of interferometers: Mach-Zehnder and Michelson interferometers. The latter's active length is twice as long as the first one, and the latter's frequency bandwidth is twice as short as the first one. There are two types of interferometric detection methods:

- Homodyne interferometric detection: The detected interferometric beat signal on the photodetector analyzed in the Fourier-domain is covered by the detector white and flicker noise around the DC signal [29].
- Heterodyne interferometric detection: The one arm in the interferometer is frequency shifted by an (acousto-optic) modulator (AOM), producing a frequency shifted signal Δ*f*_AOM_ (in the order of ≈ 10 MHz) controlled by an ultrastable RF reference. The white and flicker noises are no longer hidden by the noise from the photodetector. The self-heterodyne approach has better performance than the homodyne approach [30].

The temperature changes are represented by the relative length changes of thermal expansion of the glass of 5 × 10^−7^K^−1^, but more critically, to the thermo-optic effect, which could lead up to 10^−5^ K^−1^ [31]. On the other hand, these effects are observed on a long-term basis. Air pressure changes have a more complex effect and are more difficult to interpret. Thus, the typical fiber spool must be set into the environmentally constant conditions, blocking any ambient air changes [24]. Moreover any vibrational effects should be reduced by using the proper design and material for the spool support basis and passive and active anti-vibrational devices [32].

### 2.2. Experimental Set-Up

**Figure 1.**
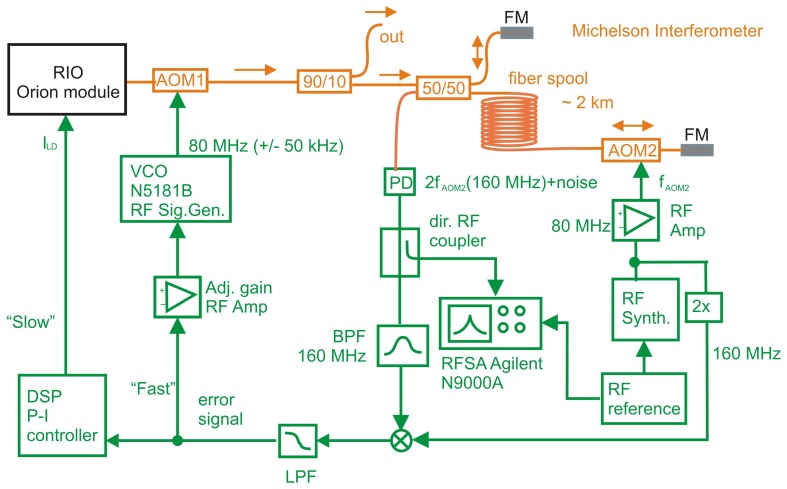
Scheme of the unbalanced interferometer for frequency stabilization: RIO (Redfern Integrated Optics) ORION^TM^ module; laser module, 90/10 and 50/50 fiber splitters; FM, Faraday mirror; AOM1, AOM2, acousto-optic modulators; VCO, voltage control oscillator; PD, photodetector; DSP, digital signal processor; LPF, low-pass filter; BPF, band-pass filter; RFSA, RF signal analyzer.

The frequency noise suppression set-up is in Figure 1. It is the Michelson fiber interferometer with the heterodyne detection scheme. The laser under test was a single-mode ORION^TM^ laser module (by Redfern Integrated Optics, Inc., Santa Clara, CA, USA) working at 1540.57 nm.

The optical part (orange lines) was built on the Michelson unbalanced heterodyne interferometer with one 2090 m-long arm. The ORION^TM^ laser module by Redfern Integrated Optics (RIO), Inc., working at a central wavelength of 1540.577 nm with a 2-kHz Lorentzian linewidth [14] and up to a 100-MHz modulation depth at a 10-kHz frequency for a 3.65-V sweep voltage, was coupled to the polarization maintaining (PM) fiber with an Angled Physical Contact (APC) fiber connector. An additional acousto-optic modulator (AOM1) that was set just in front of the the ORION^TM^ module output was used for fast detuning of the optical frequency up to the control bandwidth of the laser at *f*_1_. A driving 80-MHz signal for AOM1 was generated by the voltage control oscillator (VCO) formed by the RF signal generator, N5181B, working in the frequency modulation regime. Then, light from the laser was split into two on the 90/10 fiber splitter, where 90% of the optical power was the stabilized output port of the laser and 10% of the optical power entered the fiber Michelson interferometer. The fiber interferometer was made from single-mode fibers. The fibers had a primary protective layer of 250 *μ*m, while the part outside the 2.09 km-long spool was coated with another protective 900-*μ*m layer.

The unbalanced fiber Michelson interferometer consists of a 50/50 2 × 2 single mode fiber splitter, one short arm and one long arm. One input of the 2 × 2 splitter is input from the laser source. One of the outputs from the splitter consists of a short single-mode fiber and ends with the Faraday mirror (FM). Another output port consists of a 2.09 km-long single-mode fiber spool, a second acousto-optic modulator (AOM2) and another FM. The 2.09 km-long spool was placed into a temperature and vibrational isolated concrete box with a wooden case. The second acousto-optic modulator (AOM2) was working at a fixed frequency of 80 MHz generated by the RF synthesizer controlled by the 10-MHz RF clock signal from GPS-disciplined thermally-stabilized crystal oscillator (TSCO). The laser frequency of the light in the long arm was shifted twice by 80 MHz on AOM2, the first time on the forward route and the second time on the reflected route from the FM. The total shift of the laser optical frequency in the long arm was 160 MHz. Both the light from the long arm and from the short arm of the Michelson interferometer interfered on the 2 × 2 50/50 fiber splitter, and the interference beat signal is observed on the photodetector at the second input port of the 2 × 2 50/50 splitter. The beat frequency of 160 MHz contained information about the laser frequency change within the range of the frequency window represented by the highest frequency for delay *τ* = 20.4 *μs* (Equation (1)) on the 2.09 km-long arm of the Michelson interferometer *f*_1_ = 1/*τ* ≈ 48.8 kHz (Equation (3)). In other words, any frequency changes within this frequency range that will project onto the photodetector might be analyzed.

Servo-loop electronics: The electronic system and servo loop for the suppression of the frequency of the laser is green in the set up in Figure 1. AOM2 was fed by 80 MHz generated by the RF synthesizer controlled by the 10-MHz RF clock signal from the GPS-disciplined thermally-stabilized crystal oscillator (TSCO). The 80-MHz signal was multiplied by a multiplier generating a 160-MHz reference signal. The beat note signal from the interferometer on the photodetector PD was sent to the RF signal analyzer (RFSA), Agilent N9000A, referenced to the same 10-MHz RF clock reference. The beat note signal was filtered by the bandpass filter and mixed with the 160-MHz signal from the multiplier. The resulting mixed signal was low pass filtered and used as the input on the laser servo loop. Our servo-loop electronics consists of two arms: the “slow” servo-loop arm controlling the laser module current and the “fast” servo loop, represented by AOM1 fed by the voltage-controlled oscillator (VCO). The digital signal processor (DSP) in the “slow” arm works as a proportional-integral (PI) controller [33], processing the error signal and controlling the laser module current. The PI control speed in the “slow” arm was limited by the bandwidth of the current modulation input of the laser module, which varied between 10 and 100 kHz, depending on the signal amplitude [14]. That was the reason for using the additional P “fast” servo arm with the modulation of AOM1. The frequency modulation of the AOM1 is limited only by the maximum detectable Fourier frequency of the frequency noise of the interferometer (Equation (3)). Thus, the frequency of this AOM1 is controlled by VCO, represented by the N5181B RF signal generator in frequency modulation (FM) mode, which is fed from the RF error signal via the adjustable gain RF amplifier.

## 3. Results and Discussion

### 3.1. Single Sideband Frequency Noise Analysis

**Figure 2.**
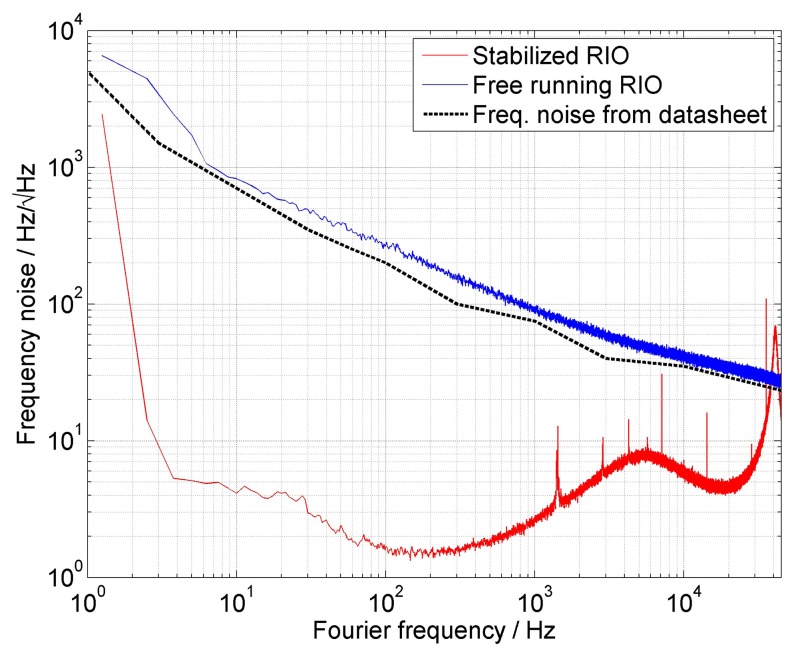
Single sideband frequency noise analysis of the ORION laser module. Comparison of the free-running, stabilized laser and the noise data from the manufacturer's datasheet [14].

The frequency noise data of the laser were recorded and collected from the Fourier frequency signal (or single sideband frequency noise (SSB)) on the RFSA, and then, they were analyzed on a computer. The Fourier spectra were normalized with respect to the *T*(*f*) function in Equation (2) and then set to the plot in Figure 2. The blue curve represents the free running laser in the loop without servo-loop control. These data were compared to the original data from the manufacturer represented by the dashed black line. As we can see, the measured noise is slightly higher than the official data from the manufacturer. This could be caused by the different systematics in the measurement and also by the additional noise in the interferometric system. The red curve represents the data for the laser in the servo loop control with the “fast” and “slow” arm presented in the set-up (Figure 1). The “slow” arm of the servo loop controls the current of the ORION^TM^ laser module *I*_LD_ (PI). With this servo-loop control, we were able to decrease the SSB noise level by more than 40 dB for Fourier frequencies from 3 Hz to 300 Hz and by up to 20 dB for frequency offsets between 300 Hz and 20 kHz. Servo-loop control over higher frequencies was not possible, partly due to the reduced signal-to-noise ratio caused by the interferometer transfer function (Equation (2)) and partly because of the bandwidth limitation in the electronics of the laser module. The “fast” arm of the servo loop represented the AOM controlled by the RF signal generator, N5181A, in FM mode. The limit of the bandwidth of this part exactly corresponds to a value of around 48 kHz (Equation (3)).

The narrow spikes in the higher frequencies represent spikes from the electronics, including DSP and the technical noise. These narrow spikes are presented only in this part of the SSB spectrum. Narrow spikes in the low frequency part of SSB, including electrical lime noise (50 Hz and its harmonics), were not presented during measurement, because the laser was supplied by batteries. We can still observe two servo bumps corresponding to the servo-loop frequency limits. One SSB frequency limit is at 5 kHz, corresponding to the control bandwidth of the laser module. The second SSB frequency bump, corresponding to the “fast” loop, is slightly below the interferometer bandwidth limit *f*_1_ at 48.8 kHz.

### 3.2. Beat Note Frequency Spectra

**Figure 3.**
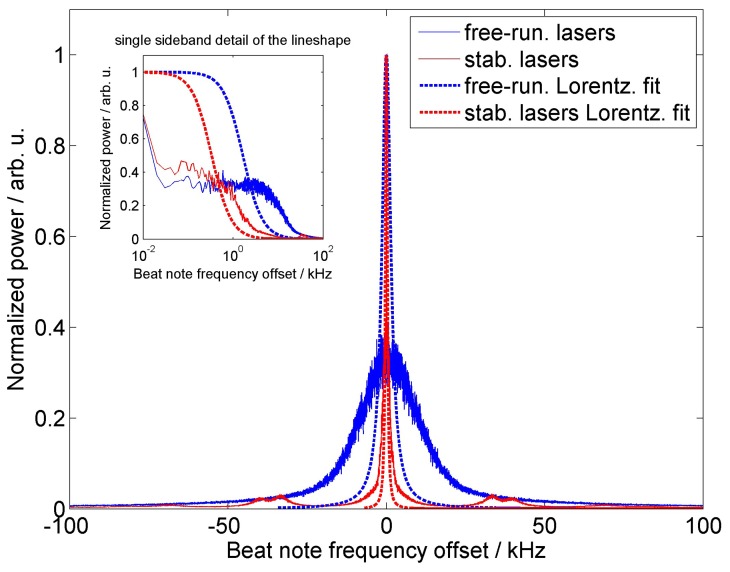
Normalized power spectra of the RF beat-note signal produced by mixing the optical outputs of two uncorrelated ORION laser modules. Free running lasers (“free-run.”) are represented by the blue line, whereas lasers stabilized to the unbalanced Michelson interferometer (“stab. lasers”) are red. Lorentzian function fits (“free-run. Lorentz. fit” for free running lasers and “stabl. lasers Lorentz. fit” for lasers stabilized to the unbalanced Michelson interferometer) for estimating the linewidth were supplemented to both datasets. Top left is the zoomed inset of the single-sideband normalized power spectra plot with logarithmic frequency axis.

Because the SSB spectra do not give information about the single frequency linewidth, We have made a deeper analysis by the two parallel set-ups (Figure 1). In our case, we made two identical noise suppression schemes for two different ORION^TM^ laser modules and made the measurement of the beat signal between both lasers. The data were collected on a digitizer card. We collected the spectra over a 10-s time period, and each scan took 0.1 s. Because both lasers had constant drift, the beat note frequency signals were overlapped over the initial one before the analysis and generation of the spectra in Figure 3.

**Figure 4.**
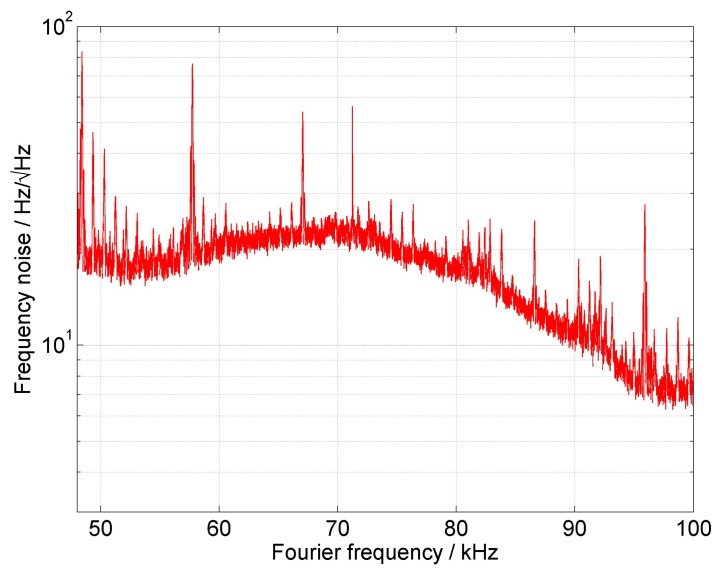
Out-of-loop measurement for stabilized and free-running ORION laser module.

The data were analyzed by the Lorentzian linewidth model to discover the laser linewidth [34]. The Lorentzian linewidth model for two free, uncorrelated ORION^TM^ laser modules working at the same wavelength show FWHM of about 1.6 kHz, as stated by the datasheet from the manufacturer [14]. On the other hand, the Lorentzian linewidth fit is not fully suitable for the manufacturer's module laser spectra. To clarify the line shape of the beat note frequency offsets for frequencies below 10 kHz, we added the inset to the top-left of the Figure 3. It shows the normalized power to a single side of the logarithmic frequency axis. We can see that the Lorentzian function fit does not correspond exactly with any beat note spectra measurement. The Lorentzian fit is the best approximation for the spectra produced by the lasers stabilized to the interferometer between 500 Hz and 25 kHz. As we can see in Figure 2, we observe in-loop white noise [29] of about $4\;\text{Hz}/\sqrt{\text{Hz}}$. According to the theory [35], one can write for the Lorentzian linewidth Δ*ν*:

(4) Δν=π⋅S(f)

where *S*(*f*) in Hz^2^/Hz is the power spectral density (PSD) of the signal noise and corresponds to the autocorrelation function of the initial signal fluctuation [29]. In the case of white frequency noise, the *S*(*f*) = *S*_0_ is constant. Therefore, we can write about 16 Hz^2^/Hz for the white noise based on the frequency noise data of the stabilized laser module. The corresponding Lorentzian laser linewidth is according to Equation (4), about 50 Hz, which is certainly below the 330-Hz linewidth. Out-of-loop measurements for Fourier frequencies *f* ≥ *f*_1_ (Figure 4) show that the white frequency noise can reach a value of about $10\;\text{Hz}/\sqrt{\text{Hz}}$; therefore, the corresponding PSD is roughly 100 Hz^2^/Hz or a 314-kHz Lorentzian fit linewidth. Although this approach gave us only rough estimates for the linewidth, it corresponds to that found by the Lorentzian fit of the beat note frequency offset.

We have observed that the beat-note spectrum for two ORION laser modules stabilized by the method presented in this paper showed decreasing of the Lorentzian profile linewidth FWHM down to 330 Hz with two sideband bumps around 40 kHz, corresponding to the results on the SSB analysis.

### 3.3. Environmental Effects

The measurement was done with the fiber spool simply placed inside the concrete box covered by a wooden box, isolating it from the air fluctuation and vibration around the fiber spool. The whole set-up was placed into the stable box, and the effects of acoustic vibrations and seismic vibrations were reduced below the limit needed by the system; thus, these effects are not expected.

The relative optical length changes with sensitivity to the temperature of 10^−5^K^−1^, with air flow drifts contributing to the low frequency noise in the Fourier SSB spectra, because they are projected on long-term drifts. For atomic and ion clock reference signals in the best time and frequency national laboratories, special care in a vacuum chamber, foam box and thermal isolation should be taken [25].

## 4. Conclusions

We have introduced the method of frequency noise suppression of the ORION^TM^ laser module based on an unbalanced heterodyne Michelson interferometer with a 2.09-km delay arm. We suppressed the single-sided band noise level by more than 40 dB for the Fourier frequency bandwidth from 3 Hz to 300 Hz and by up to 20 dB for Fourier frequencies up to 20 kHz. The linewidth beat note frequency measurement between two lasers stabilized by the same type of unbalanced Michelson interferometer showed a decrease of the linewidth from 1.5 kHz to 330 Hz.
